# The molecular biology and HPV drug responsiveness of cynomolgus macaque papillomaviruses support their use in the development of a relevant in vivo model for antiviral drug testing

**DOI:** 10.1371/journal.pone.0211235

**Published:** 2019-01-25

**Authors:** Eva-Maria Tombak, Andres Männik, Robert D. Burk, Roger Le Grand, Ene Ustav, Mart Ustav

**Affiliations:** 1 University of Tartu, Institute of Technology, Tartu, Estonia; 2 Icosagen Cell Factory Ltd., Eerika tee 1, Õssu, Kambja, Tartumaa, Estonia; 3 Department of Epidemiology & Population Health, Albert Einstein College of Medicine and Montefiore Medical Center, Bronx, New York, United States of America; 4 Department of Pediatrics (Genetics), Albert Einstein College of Medicine and Montefiore Medical Center, Bronx, New York, United States of America; 5 Department of Microbiology & Immunology, Albert Einstein College of Medicine and Montefiore Medical Center, Bronx, New York, United States of America; 6 Department of Obstetrics, Gynecology & Women's Health, Albert Einstein College of Medicine and Montefiore Medical Center, Bronx, New York, United States of America; 7 CEA, Université Paris Sud, INSERM U1184, Immunology of Viral Infections and Autoimmune Diseases (IMVA), IDMIT Department / IBFJ, Fontenay-aux-Roses, France; 8 Estonian Academy of Sciences, Tallinn, Estonia; Penn State University School of Medicine, UNITED STATES

## Abstract

Due to the extreme tissue and species restriction of the papillomaviruses (PVs), there is a great need for animal models that accurately mimic PV infection in humans for testing therapeutic strategies against human papillomaviruses (HPVs). In this study, we present data that demonstrate that in terms of gene expression during initial viral DNA amplification, *Macaca fascicularis* PV (MfPV) types 5 and 8 appear to be similar to mucosal oncogenic HPVs, while MfPV1 (isolated from skin) resembles most high-risk cutaneous beta HPVs (HPV5). Similarities were also observed in replication properties during the initial amplification phase of the MfPV genomes. We demonstrate that high-risk mucosal HPV-specific inhibitors target the transient replication of the MfPV8 genomes, which indicates that similar pathways are used by the high-risk HPVs and MfPVs during their genome replication. Taking all into account, we propose that *Macaca fascicularis* may serve as a highly relevant model for preclinical tests designed to evaluate therapeutic strategies against HPV-associated lesions.

## Introduction

Human papillomaviruses (HPVs) are medically important pathogens that are responsible for a diverse range of epithelial manifestations ranging from asymptomatic infections to malignant neoplasia. Papillomaviruses are tissue-specific viruses that infect epithelial cells at different anatomic locations and can be transmitted through direct contact with infected tissue. HPVs are clustered phylogenetically into five (alpha, beta, gamma, mu and nu) genera, of which the alpha and beta viruses are the most extensively studied [1]. Beta HPVs (e.g., HPV5 and 8) target cutaneous epithelia, and their infections are usually asymptomatic in healthy individuals; however, in immunocompromised patients, these viruses have been suggested as etiological factors in the development of nonmelanoma skin cancer (NMSC) [2–5]. The members of the alpha genus infect mucosal epithelium and can be subdivided into high- and low-risk HPVs (HR-HPV and LR-HPV, respectively) based on their association with malignant progression [6]. Low-risk HPV types (e.g., HPV6 and HPV11) mainly cause genital warts, condylomas and papillomas that generally do not progress into malignant tumors [7]. In most instances, HR-HPV infections occur without any symptoms and are readily cleared by the immune system. Nevertheless, in some cases, HR-HPV (e.g., HPV16, 18, 31, 45) infections persist, causing genetic instability that can lead to the development of several anogenital (anal, penile, vulvar and vaginal) and head and neck cancers [8,9]. Cervical cancer is the fourth most common cancer among women worldwide; approximately half million new cases of malignant tumors are diagnosed each year, leading to death in approximately half of the cases [10]. The epidemic increase in HPV-positive oropharyngeal squamous cell carcinomas (OPSCC) has been reported recently–now up to 85% of the cases in the US and an even higher frequency in some European countries are HPV-positive [11–13]. Regardless of causing serious health problems and economic burden, there is still no approved effective cure for an ongoing HPV infection. Although there are approved invasive treatments removing the infected area in a quite extensive manner, such as cryogenic destruction, larger excision procedures, laser therapy and electrosurgery, these approaches do not eliminate HPV infection completely and therefore often lead to the necessity of repeated treatments (approximately a 40% chance of recurrence) [14,15]. Additionally, three efficacious prophylactic vaccines against HPV are available on the market: Gardasil (against types 6, 11, 16 and 18), Gardasil 9 (against types 16, 18, 31, 33, 45, 52, 58, 6 and 11) and Cervarix (against types 16 and 18); however, these vaccines target only nine of the most common HPV types and are restricted to those individuals who are naïve to certain HPV types [16,17]. Therefore, there is a great need for antiviral agents for treating ongoing HPV infections.

HPV drug candidates should be tested in in vivo animal models for their safety and efficacy. Animal models for animal papillomaviruses have been in place for a long time and work effectively for murine, canine, bovine and rabbits PV models. However, current preclinical models have a number of limitations that are linked to the fact that HPVs replicate and complete their life cycle only in differentiating human keratinocytes and cause cancers at discrete epithelial sites that are not straightforward to model in vivo [18]. Widely used models such as cottontail rabbit papillomavirus and canine oral papillomavirus differ from oncogenic HPVs in terms of genetic organization, gene expression, life cycle dynamics, genital mucosal tropism, and persistence [19]. Thus, there is a great need for animal models that accurately mimic PV infection in humans. Nonhuman primates captured from wild are quite frequently infected with specific papillomaviruses, and can cause clinical manifestations of productive viral infection, similar to HPV-associated lesions in the human skin and anogenital region [1,20–25]. *Macaca fascicularis* PVs are phylogenetically and genetically very close to human high-risk as well cutaneous papillomaviruses [1,20,21,23–25].

In the present study, we assembled *Macaca fascicularis* papillomavirus genomes MfPV1 (beta type) [21], MfPV5 and MfPV8 (high-risk types) [23] and characterized their replication properties and transcription pattern in human U2OS cells using 5’ and 3’ RACE analysis and RT-PCR. The U2OS cell line derived from a moderately differentiated osteosarcoma has a unique capability to support the transient, stable, and late amplificational replication of both cutaneous and mucosal HPV genomes efficiently [26]. The replication and transcriptome studies suggest that U2OS cells provide an adequate cellular environment to study HPV [26–32]. It has been shown that the same HPV DNA replication intermediates observed in U2OS cells are also present in keratinocyte cell lines [26,28,33]. Additionally, transcriptome analyses of mucosal HPV types 11 and 18 and cutaneous HPV5 have been published [29–31], indicating that HPV gene expression in U2OS cells is characteristic of HPV-positive keratinocytes [34,35].

In this study, we present data that demonstrate that the gene expression of mucosal high-risk MfPV5 and MfPV8 is characteristic of mucosal oncogenic HPVs, while MfPV1 appears to be similar to high-risk cutaneous beta HPVs (HPV5). Similarities were also observed in replication properties during the initial amplification phase of the MfPV genomes. The analysis of uncut MfPV DNA revealed that multimerization of viral genomes, which is common to HPVs, is also a characteristic feature of MfPV DNA replication. Moreover, the replication of the MfPV8 genomes was sensitive to mucosal high-risk HPV-specific inhibitors, indicating that similar pathways are involved in high-risk HPV and MfPV genome replication. Taken together, these molecular findings suggest that *Macaca fascicularis* could be a promising animal model system for verification of the therapeutic effects of anti-HPV drug candidates.

## Materials and methods

### Cell lines and transfection

U2OS cells (American Type Culture Collection, ATCC no: HTB-96), were grown in Iscove's modified Dulbecco's medium (IMDM) supplemented with 10% fetal calf serum. U2OS cells were transfected by electroporation using a Bio-Rad Gene Pulser II apparatus equipped with a capacitance extender (Bio-Rad Laboratories) at 220V, and capacitance set to 975μF [36,37].

### Plasmids

To produce plasmid-free covalently closed circular MfPV genomic DNA (referred to as minicircles) with the minor 43-44-bp remnant of the recombination site inserted into the MfPV genomic sequence [38], the minicircle parental plasmids pMC-MfPV1, pMC-MfPV5, and pMC-MfPV8 were engineered. For this purpose, PCR reactions were performed using the MfPV5 or 8 genomic DNA fragments [23] as template DNA to generate viral PCR fragments bearing overhangs of desired homology to the flanking regions. Using the modified Gibson assembly method [39,40], the viral fragments and the linearized pMC.BESPX vector [38] were assembled. The resulting plasmids pMC-MfPV5 and pMC-MfPV8 had the vector backbone in positions between nt 7534 and nt 7535 in the MfPV5 genome (according to the GenBank ID EF558843) and between nt 7558 and nt 7559 in the MfPV8 genome (GenBank ID EF558842), respectively. For MfPV1, the viral genome was obtained as synthetic DNA used to construct pMC-MfPV1, and the minicircle parental vector backbone was inserted into the MfPV1 genome (GenBank ID EF028290) between nt 7334 and nt 7335. The following methods were used to engineer MfPV genomic mutants from the MfPV minicircle parental plasmids: for cloning the E8 mutant genomes, the ATG start codon of E8 ORF was mutated to ACG (at position 1074, 1202, 1163 in the MfPV1, 5, and 8 genomes, respectively) because it does not change the amino acid sequence of the overlapping E1 ORF. The viral E1 or E2 defective genomes were engineered by introducing two consecutive in-frame stop codons and a unique endonuclease recognition site at the beginning of the respective ORF. The E1 or E2 mutant genomes of MfPV1 and MfPV5 were generated in the context of E8 mutant genomes; for MfPV8, the wild-type genome was used. For the MfPV1 E1 mutant, the nucleotides CAAGCTTTGATAGTCACTACAAGAGCT (HindIII) were added after genomic position 887; for the MfPV1 E2 mutant, the nucleotides CGATCGGATCCAGTGATAGTCATCAAT (BamHI) were added after genomic position 2549; for the MfPV5 E1 mutant, the nucleotides GTATTGATAAGCTTA (HindIII) were added after genomic position 844; for the MfPV5 E2 mutant, the nucleotides CATGTAGTGAGAGCTC (SacI) were added after genomic position 2702; for the MfPV8 E1 mutant, the nucleotides CTAGGGTTGAGCTCTGATAGTCATCAC (SacI) were added after genomic position 2311; for the MfPV8 E2 mutant, the nucleotides CCAGGGCTAGTGAGATCTTCACTATGA (BglII) were added after genomic position 2784. The viral minicircle genomes were produced following the previously published protocol [36].

### Chemicals

The compounds NSC9782, NSC 88915, NSC 82269, NSC 109128 and NSC 305831 were obtained from the Drug Synthesis and Chemistry Branch, Developmental Therapeutics Program, Division of Cancer Treatment and Diagnosis, National Cancer Institute.

### Transient replication assay

For transient replication assay of MfPV genomes, U2OS cells were transfected with 4–5 μg of the indicated MfPV minicircle genomes. Total genomic DNA [26] or low-molecular-weight (LMW) DNA was extracted at the indicated time points using the modified Hirt method [36,41]. Prior to analysis half of each extrachromosomal DNA sample extracted from a 100 mm cell culture dish or 5 μg of each total DNA sample was treated with specific endonucleases linearizing viral DNA and DpnI (Thermo Scientific) to remove bacterially produced input MfPV DNA. Extracted DNA molecules were resolved in a 0.8% agarose gel in TAE (1× Tris-acetate-EDTA) buffer (0.8 V/cm for 20 h) and visualized via Southern blotting (previously described in ref. [36]). Hybridization probes were prepared from specific MfPV genomic fragments using the DecaLabel DNA Labeling Kit (Thermo Scientific) and radioactive [α-32P] dCTP (Hartmann Analytic). The resultant signals were detected via exposure to X-ray (Fuji) or using Amersham Typhoon RGB (GE Healthcare). The experiments were performed 2–3 times.

### RNA extraction, rapid amplification of cDNA ends (RACE) and RT-PCR

PolyA+ RNA was extracted at the fourth day time-point from U2OS cells transfected with a MfPV minicircle genomes. The RNA samples were prepared by isolation of total RNA by TriReagent (Molecular Research Center, Inc.) followed by polyA+ RNA purification using NucleoTrap mRNA Mini kit (MACHEREY-NAGEL GmbH) according to the manufacturer’ instructions. 1 μg of polyA+ RNA was used as a template for 5' or 3' RACE, respectively, performed with the SMARTer RACE 5’/3’ Kit (Takara Bio USA, Inc.) according to the manufacturer's instructions. For RT-PCR, 1 μg cDNA first-strand synthesis was conducted using the SuperScript IV First-Strand Synthesis System (Thermo Fischer Scientific, USA). The Phusion Hot Start II DNA polymerase (Thermo Fischer Scientific, USA) was used to amplify the RACE and RT-PCR products that were purified from agarose gel, cloned and fully sequenced as single clones (data in S1 and S2 Tables). The positions of the MfPV genome specific primers (GSPs) used for the RACE and RT-PCR amplifications are listed in Table 1.

**Table 1:** Sequences of primers used for RACE analyses and RT-PCR.

	Biding position	Sequence (5’→3’)
**MfPV5 (EF558843)^a^**	3391–3366 C	CAGCAGAATCTGGTCTGCGTCGCTTG
	1313–1287 C	CCTGTCCTCTTCCGGCTGCCTATTCTC
	3845–3870	GCTAAGCCTGTAAACCGCCACAATTC
	3366–3391 C	CAGCAGAATCTGGTCTGCGTCGCTTG
	1068–1089	GTAAGCACTCCAACGGTTAGTC
**MfPV8 (EF558842)^a^**	3404–3378 C	AGCAGTGCGTTGCTGTTATGGTCCAAG
	1294–1268 C	ATGCTCTCCTGCGAACCCTCCTCAATC
	84–108	GCACTGCGTGTATTGCAGGGAAGAG
	3853–3879	GTGCTGCTAGGCCTGTAAACCGCAACC
	1157–1176	CGGCTATGGCAATACTCAAG
	3378–3404 C	CGGCTATGGCAATACTCAAG
**MfPV1 (EF028290)^a^**	3187–3161 C	CCTGGTGTTGGCTTCGGATGATTGTTG
	711–684 C	CGCCATTGGGCTGTAGATGTGCTCTTCG
	872–847 C	CCCTGCTCCAGGTCTGCATTATCAAC
	1064–1039 C	GATAGTTCTAGCCCGCTGTCTTGTTC
	3848–3874	CGTCTAGGCAGGTCCAGAATGCTCATC
	862–885	CTGGAGCAGGGAAATTCCCGAGAG
	3161–3187 C	CCTGGTGTTGGCTTCGGATGATTGTTG

^a^ GenBank ID

## Results

### Macaque papillomavirus genomes can replicate in the human U2OS cell line

Zigui Chen and colleagues managed to isolate and characterize 10 distinct types of PVs from cervical tissue from different populations of female *Macaca fascicularis* originating from China and Indonesia. Four MfPVs (types 3, 4, 5 and 8) were detected in CIN lesions and should be considered high-risk types. Sequence comparisons and phylogenetic analyses clustered these identified MfPVs within the alpha PV α12 species group closely related to the α9 (e.g., HPV16) and α11 species (e.g., HPV34) [23]. *M*. *fascicularis* papillomavirus type 1 (MfPV1) was identified in severe cutaneous papillomas on the hands and feet of a cynomolgus macaque and clustered into the genus betapapillomavirus [21].

**Fig 1:**
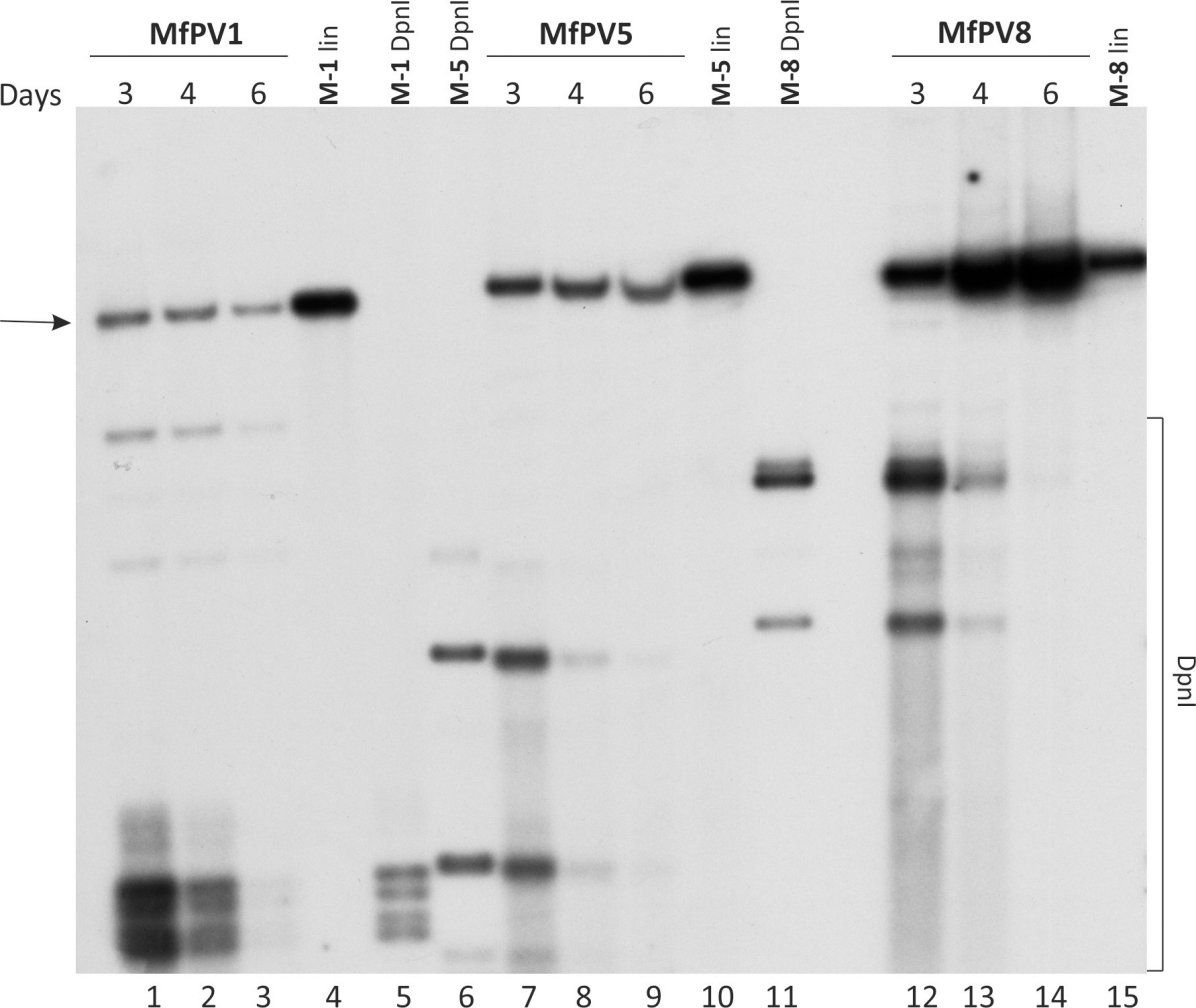
The transient replication of the MfPV1, 5 and 8 genomes in U2OS cells. U2OS cells were transfected with 5 μg of MfPV1 (lanes 1–3) and 4 μg of MfPV5 (lanes 7–9) and 8 (12–14) minicircle genomes. Low molecular weight (LMW) DNA was extracted at the indicated time points (days) and analyzed through 1D gel electrophoresis, followed by Southern blotting. Prior to analysis, the samples were treated with DpnI and specific linearizing endonuclease (SdaI for MfPV1, BamHI for MfPV5, and BglI for MfPV8) (Thermo Scientific). Lanes 4, 10 and 15 are size markers for linear MfPV1, 5 and 8 DNA, respectively. Lanes 5, 6 and 11 are size markers for the DpnI fragments of MfPV1, 5 and 8 genomes, respectively. DpnI, the fragments of DpnI-digested unreplicated genomes. Arrow marks the linear MfPV genomes.

We demonstrated that the human osteosarcoma cell line U2OS supports the genome replication of alpha and beta HPVs and is suitable for studying HPV genome replication and transcription [26–32,42]. To identify whether the same cell line also supports the replication of the MfPV genomes, a transient replication assay mimicking the phase of the establishment of the viral infection was performed. Using the modified Gibson assembly method [39,40], the genomic fragments of different *Macaca fascicularis* alphapapillomavirus types, MfPV5 and MfPV8, and a betapapillomavirus type, MfPV1, were cloned into the pMC.BESPX minicircle producer vector [38], as described in Materials and Methods. The minicircle technology provides an efficient and cost-effective system for the production of bacterial backbone-free covalently closed circular (ccc) DNA molecules (referred to as minicircles for the brevity) that are similar to the native viral genomes [38]. The MfPV genomic minicircles were transfected into U2OS cells by electroporation. At the indicated time points, low-molecular-weight (LMW) DNA was extracted from the cells using the Hirt lysis protocol [41], subsequently purified, and analyzed through Southern blotting after linearization with specific endonucleases and digestion with DpnI to remove bacterially produced input DNA. Three distinct radiolabeled probes (MfPV1, 5 and 8 genomic DNA probes) were used to selectively analyze the replication of specific MfPV genomes. These results indicated that the U2OS cell line effectively supports MfPV DNA replication (Fig 1, lines 1–3, 7–9, 12–14). Both MfPV8 and MfPV5 genomes showed the DpnI-resistant signal accumulation in U2OS cells. While the MfPV8 genome copy number obviously increased over time (Fig 1, lines 12–14), the replication signal of MfPV5 slightly decreases at later time points (Fig 1, lines 7–9). The beta-PV MfPV1 genomes clearly replicated in U2OS cells; however, the intensity was rather modest (Fig 1, lines 1–3) compared to the alpha subtypes (Fig 1, lines 7–9, 12–14). The same observations have also been noted among human betaPVs (e.g., HPV8) in the U2OS cell line [26]. In conclusion, the results indicate that U2OS cells provide an adequate cellular environment to study the molecular mechanisms not only for a variety of HPVs but also for *Macaca fascicularis* PVs.

### Construction of a transcription map of MfPV1, 5 and 8 during transient replication in U2OS cells

Based on the sequence level prediction, the gene expression pattern characteristic for the specific HPV and MfPV types seems to be very similar ([21,23], http://pave.niaid.nih.gov). As the transient replication assay revealed that MfPV genomes are capable of initiating DNA replication in the U2OS cell line (Fig 1), it implies that MfPV genomes are transcriptionally active in these cells, thus providing the tool for experimental description of the viral transcriptomes. Thus, polyA+ RNA was extracted at the 4th day time point from U2OS cells transfected with MfPV1, 5 or 8 minicircle genomes. In this timeframe, the replication of viral DNA was clearly initiated (Fig 1); thus, these samples represented the initial amplification phase of the viral DNA. As described in the Materials and Methods, we used extracted RNA to perform 5′ and 3′ RACE analyses and RT-PCR to identify the viral transcription start sites (TSSs), splice junctions and polyadenylation cleavage sites (CSs) in the viral early region for MfPV types 1, 5 and 8. The sequences of all primers used and their positions in the MfPV genomes are given in Table 1.

The results revealed that for MfPV types 5 and 8, the most predominantly used early promoter region located within the long control region (LCR), the TSS mapped immediately upstream of the E6 gene (at nt 7982 and 7996, respectively) (S1 and S2 Figs), and a canonical TATA box element was also identified [23]. These findings are consistent with the early TSSs found upstream of the E6 ORF in several HPVs, such as P97 for HPV16 [43,44] and P102 for HPV18 [29,35,45–47]. Additional TSSs positioning in the E6, E7, E1 and E2 ORFs were detected (S1 and S2 Figs). Similar promoter regions have also been described for HPV18 in U2OS cells and in other studies ([29] and references therein). In contrast to alpha MfPV5 and 8, beta-PV MfPV1 showed more heterogeneity in TSS usage because highly predominant TSS was not detected (S3 Fig). To compare the results with beta HPV type 5, similarities can be found in TSS usage; for instance, a strong promoter region and multiple TSSs within the E7 ORF have also been described for HPV5 by Sankovski and others [30].

The mapping of the viral transcripts expressed from the mucosal MfPV5 and 8 genomes in U2OS cells revealed several splicing donor (SD) (at nt 123, 819, 1235 for MfPV5 and nt 777, 1037, 1196 for MfPV8) and acceptor (SA) sites (at nt 341, 2588, 3237 for MfPV5 and nt 2570, 3219 for MfPV8) (S1 and S2 Figs), and clear sequence homology was noticed with the corresponding SD and SA sites of human high-risk types 16 and 18 [29,35,43,48]. A common future of high-risk HPVs associated with malignant progression (e.g., HPV16, 18, 31, 45) is that E6/E7 polycistronic mRNA has a splice donor and acceptor pair in the E6 ORF that enables the expression of shorter forms of the E6 proteins (termed E6*) and facilitates more effective translation of the E7 oncoprotein [49–51]. The sequencing of MfPV5 DNA revealed the presence of this canonical E6 SD site within the E6 ORF [23], and the usage of this SD for the generation of viral mRNAs was confirmed by 5’ RACE analysis (S1 Fig, RNAs B-D, G). In contrast, regardless of the association of MfPV8 with CIN lesion [23], a mutation in the core motif of the E6 ORF containing the SD site (AG^GT → AT^GT) is described ([23], GenBank ID EF558842); indeed, we did not detect transcripts spliced inside of the E6 ORF (S2 Fig, RNAs A-D). Interestingly, the putative splice acceptor site in the E6 ORF of MfPV5 was predicted at nucleotide 306 by sequence homology analyses; however, no transcripts were identified utilizing this splice acceptor site; the SA site at nucleotide 341 was used instead (S1 Fig, RNAs B-D). In the case of MfPV1, identified splice donor (at nt 721, 1037, 1101) and acceptor (at nt 2412, 3058, 3219) sites also exhibit sequence homology in the SD and SA regions of closely related HPV5 [30] (S3 Fig). The coding potential is described to the right of each transcript in S1–S3 Figs. In summary, the identified viral mRNA species for all analyzed MfPVs can be templates for translation of most, if not all, PV early proteins.

The functional polyadenylation CSs of early mRNAs of all three MfPVs localized near the beginning of the L2 ORF (approximately nt 4310 for MfPV5, nt 4300 for MfPV8, and nt 4185 for MfPV1) (S1–S3 Figs, respectively) with a conserved polyadenylation motif AAUAAA found closely upstream. The identified CS regions exhibited sequence homology to the analogous regions of closely related HPVs, most conspicuously for MfPV1, which was highly homologous to preferred early CSs identified in HPV5 [29,30,35]. Generally, based on transcriptional analysis, MfPV5 and 8 appear to be similar to mucosal oncogenic HPVs, while MfPV1 appears to be similar to high-risk cutaneous HPVs.

### E8 ORF is expressed in MfPVs and is involved in the negative regulation of MfPV DNA replication

Papillomaviruses encode alternative E2 proteins acting as transcription regulators that control the expression levels of different viral transcripts and thus regulate viral genome copy number. Our MfPV transcriptome mapping revealed the transcripts with the capability to encode small (10–12 aa) E8 peptide-containing proteins of the MfPV1 (S3 Fig, RNAs D and E), 5 (S1 Fig, RNAs J and K) and 8 (S2 Fig, RNAs G and H) during transient replication in U2OS cells. All E8 transcripts are generated by splicing at the E8 SD site located in the E1 ORF but can be combined with different SA sites located in either the E1 or E2 ORF. Thus, for all three MfPVs, we identified mRNA species that have the capability to encode either E8^E1 or E8^E2 polypeptides, respectively. In the predicted E8^E1 protein, the E8 peptide is in-frame fused with the very C-terminal part of the E1 protein, and for several HPVs (e.g., types 18, 11, and 5), it was demonstrated that E8^E1 has no significant role in transient replication [29–31]. In E8^E2, the E8 peptide is in-frame fused with the C-terminal DNA binding domain of the E2 protein. In contrast to E8^E1, the E8^E2 protein has been shown to be the major negative regulator of viral transcription and DNA replication, which has been previously described in several human and animal papillomaviruses (e.g., BPV1, CRPV) [52–58].

**Fig 2:**
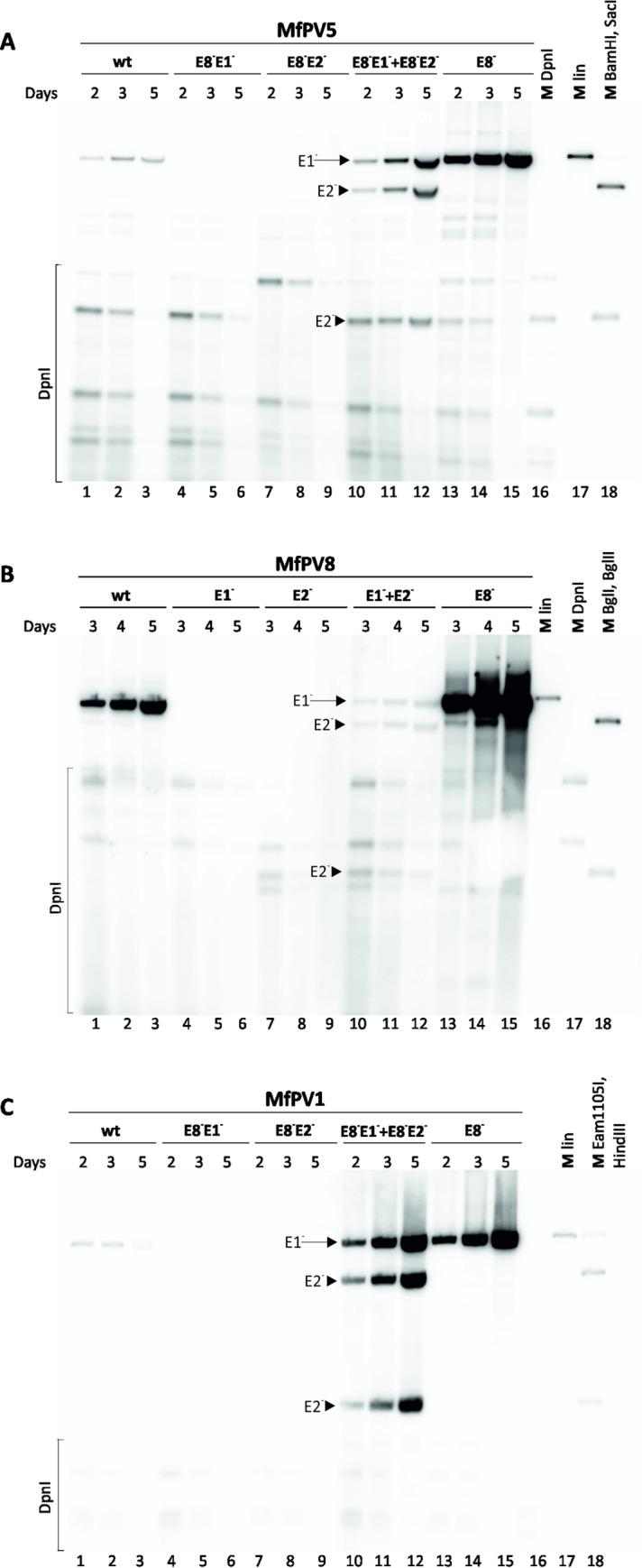
Transient replication analysis of the MfPV wild-type and mutant genomes in the U2OS cell line. U2OS cells were transfected with 4 μg of wild-type (wt) or mutant minicircle genomes of MfPV5 (A), MfPV8 (B), and MfPV1 (C) defective in E1 (E1-), E2 (E2-) and/or E8^E2C (E8-) expression. For the complementation assay, the cells were cotransfected with E1- and E2-defective genomes (4 μg each). Episomal DNA was extracted via Hirt lysis at the indicated time points (days) and subjected to Southern blotting. Prior to analysis, the samples were treated with DpnI and specific endonucleases (Thermo Scientific). (A) In the case of MfPV5, the E8-E1- and E8-E2- double mutant genomes were used. MfPV5 wt (lines 1–3), E8-E1- (lines 4–6) and E8-E2- (lines 7–9) samples were digested with the linearizing enzyme BamHI, while E8-E1- plus E8-E2- samples (lines 10–12) were double digested with BamHI and SacI (only cuts the E2 mutant genome) to facilitate distinction between cotransfected plasmids. (B) MfPV8 wt (lines 1–3), E1- (lines 4–6) and E2- (lines 7–9) samples were digested with the linearizing enzyme BglI, while E1- plus E2- samples (lines 10–12) were double digested with BglI and BglII (only cuts the E2 mutant genome). (C) In the case of MfPV1, the E8-E1- and E8-E2- double mutant genomes were also used. MfPV1 wt (lines 1–3), E8-E1- (lines 4–6) and E8-E2- (lines 7–9) samples were digested with the linearizing enzyme Eam1105I, while E8-E1- plus E8-E2- samples (lines 10–12) were double digested with Eam1105I and HindIII (only cuts the E1 mutant genome). DpnI-treated, linear and double digested marker plasmids for MfPV5, MfPV8 or MfPV1 are shown in lanes 16 to 18. In the case of complementation assay, linear viral genome is indicated with arrows, double digested genome fragments with arrowheads. Abbreviations: E1-, E1 mutant genomes; E2-, E2 mutant genomes.

To study whether the E8 ORF (most likely in composition of E8^E2) is also involved in the control of MfPV replication intensity, we generated E8-defective genomes for MfPV1, 5 and 8, where the E8 start codon was mutated (described in Materials and Methods). The wild-type or mutant minicircle genomes of MfPV1, 5 and 8 were transfected into U2OS cells. LMW DNA was extracted via Hirt lysis [41] at the indicated time points and subjected to Southern blotting after linearization with appropriate endonucleases and digestion with DpnI for fragmentation of the bacterially produced input DNA. The data presented in Fig 2 indicate that the loss of the E8 initiation codon in the MfPV1, MfPV5 and MfPV8 genomes resulted in considerably more efficient replication of all three MfPVs compared with the wild-type genomes (Fig 2A and 2B, compare lanes 1–3 and 13–15), most notably for the MfPV1 (Fig 2C, compare lanes 1–3 and 13–15), suggesting that, similar to many other PVs [52–58], E8^E2 is an important transcriptional repressor involved in the initial replication of the MfPV1, 5 and 8 genomes.

### MfPV replication in U2OS cells is strictly dependent on the presence of PV replication proteins E1 and E2

Another thoroughly described phenomenon of the initiation of papillomavirus transient DNA replication is its dependence on viral replication proteins encoded by full-length E1 and E2 ORF [37,59–64]. To define the implication of the E1 and E2 proteins in the MfPV genome replication, we generated mutant genomes of the MfPV8 defective for expression of the E1 or E2 proteins as a result of the insertion of stop codons and a unique endonuclease recognition site onto the respective ORFs (E1 or E2 defective genomes described in Materials and Methods). In the case of MfPV1 and MfPV5, the E1 or E2 defective genomes were generated in the context of E8^E2 mutant genomes because of its increased replication capability that facilitates the replication analysis. The wild-type and mutated genomes alone or in combination (E1 mutant plus E2 mutant) were transfected into the U2OS cells. At the indicated days posttransfection, episomal DNA was extracted via Hirt lysis [41], and the results were visualized via Southern blotting as described above. Analysis of the LMW DNA revealed that the MfPV8 wild-type genomes (Fig 2B, lanes 1–3) and the MfPV1 and MfPV5 E8^E2 mutant genomes (Fig 2A and 2C, lanes 1–3, respectively) replicated readily in a time-dependent fashion in U2OS cells. However, a defect either in the E1 or E2 ORF eliminated the capability of the MfPV8 (Fig 2B, lanes 4–9), MfPV5 (Fig 2A, lanes 4–9) and MfPV1 (Fig 2C, lanes 4–9) genomes to replicate, while cotransfection of the E1 and E2 mutant genomes restored the replication of both genomes that was distinguished by the unique endonuclease recognition site inserted onto the E2 ORF (Fig 2A–2C, lanes 10–12). This clearly demonstrates that the two defective viral genomes complement each other’s defects, and thus we believe that our data convincingly demonstrate that the transient replication of MfPV1, MfPV5 and MfPV8 in U2OS cells is strictly dependent on the expression of both E1 and E2 proteins.

### The MfPV genomes form oligomeric structures during transient DNA replication in U2OS cells

**Fig 3:**
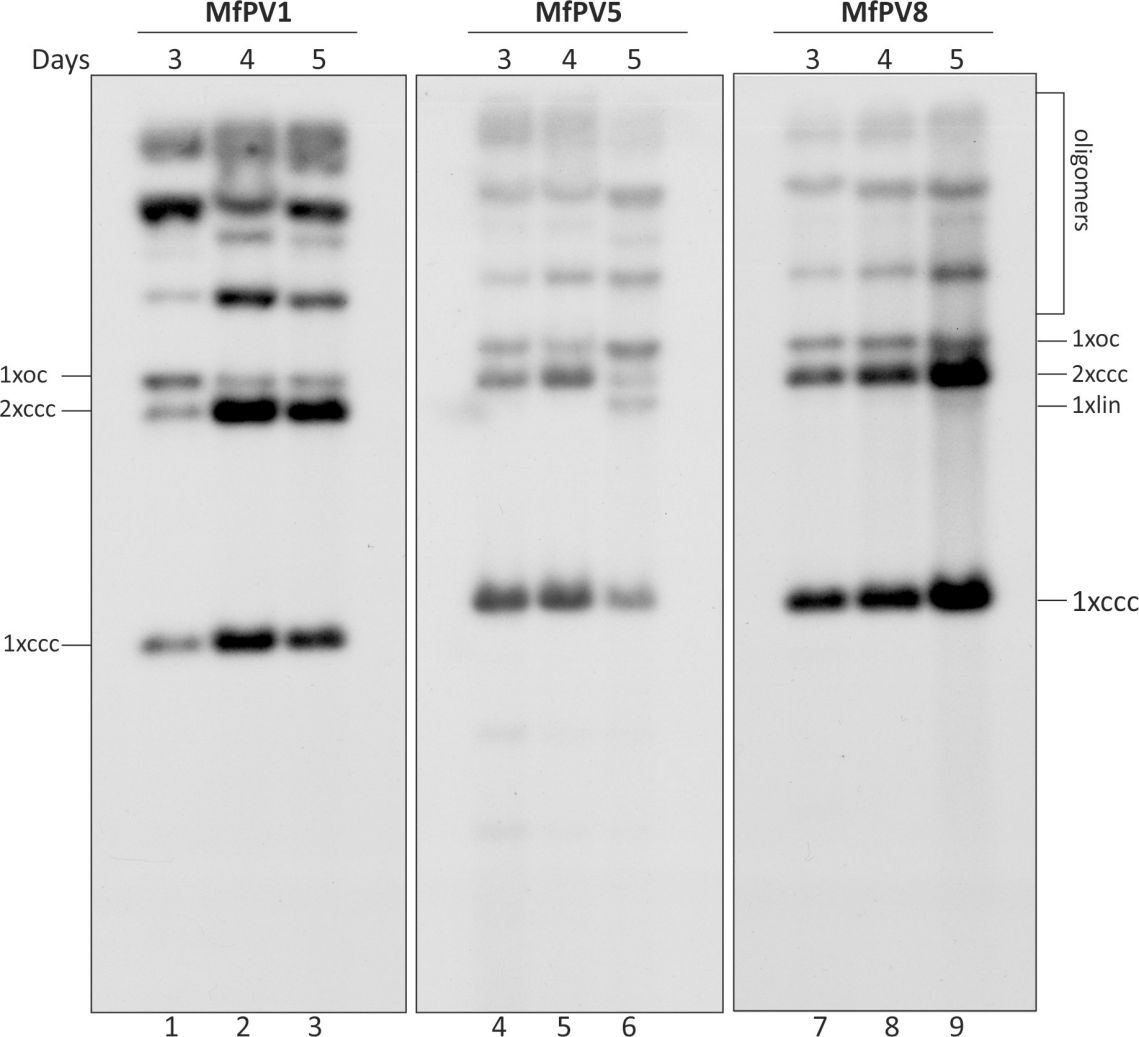
The physical state of the MfPV genomes during transient DNA replication in U2OS cells. U2OS cells were transfected with 4 μg E8^E2C mutant minicircle DNA of MfPV1 (lanes 1–3), MfPV5 (lanes 4–6), and MfPV8 (lanes 7–9). LMW DNA extracted via Hirt lysis 3, 4 and 5 days posttransfection (shown on the top), the samples were treated with DpnI, and analyzed by Southern blotting. Abbreviations: oc, open circular molecules; ccc, covalently closed circular molecules; lin, linear molecules; the numbers 1 and 2 refer to monomers and dimers, respectively.

During the initial amplification phase of HPV DNA, it has been noticed that in addition to monomeric HPV genomes (containing a single copy of the HPV genome), the viral genomic oligomers also appear [26,27,36]. Furthermore, during the stable maintenance phase, the majority of HPV DNA molecules persist in the multimeric forms in U2OS cells [26,27]. The multimeric papillomavirus genomes have also been detected in clinical samples obtained from HPV-associated cervical lesions [27,65–69], HPV-infected keratinocyte cell lines [27,70,71], and BPV1-transformed mouse fibroblast cell line ID13 [72]. To analyze whether this naturally occurring phenomenon is also characteristic of MfPV genome replication, we analyzed the physical state of viral DNA in U2OS cells. For this, the MfPV1, MfPV5 and MfPV8 E8^E2 mutant minicircle genomes were transfected into U2OS cells, and LMW DNA was analyzed through Southern blotting without linearization or fragmentation of the viral genomes; however, only DpnI treatment was performed to remove bacterially produced input DNA. Thus, the examination of viral DNA topology was performed by undigested replicated DNA forms. As shown in Fig 3, the MfPV DNA replication signal revealed a series of discrete bands. The monomeric MfPV genomes were detected in three main topological forms: supercoiled covalently closed circles (1 ccc), linear molecules (1 lin), and open circular molecules with single-strand nicks (1 oc) (Fig 3, all of which are indicated). The oligomeric genomes with higher molecular weights exhibited a retarded migration pattern in the agarose gel, and the results in Fig 3 indicate the accumulation of monomeric and much larger slower-migrating molecules over time, indicating the formation of the oligomeric genomes of all analyzed MfPVs (Fig 3). Thus, the oligomerization that is shown for many HPV genomes, including patient samples, is also characteristic of MfPV genome replication in U2OS cells.

### Inhibition of initial amplification of the MfPV8 genomes by the high-risk HPV-specific compounds

**Fig 4:**
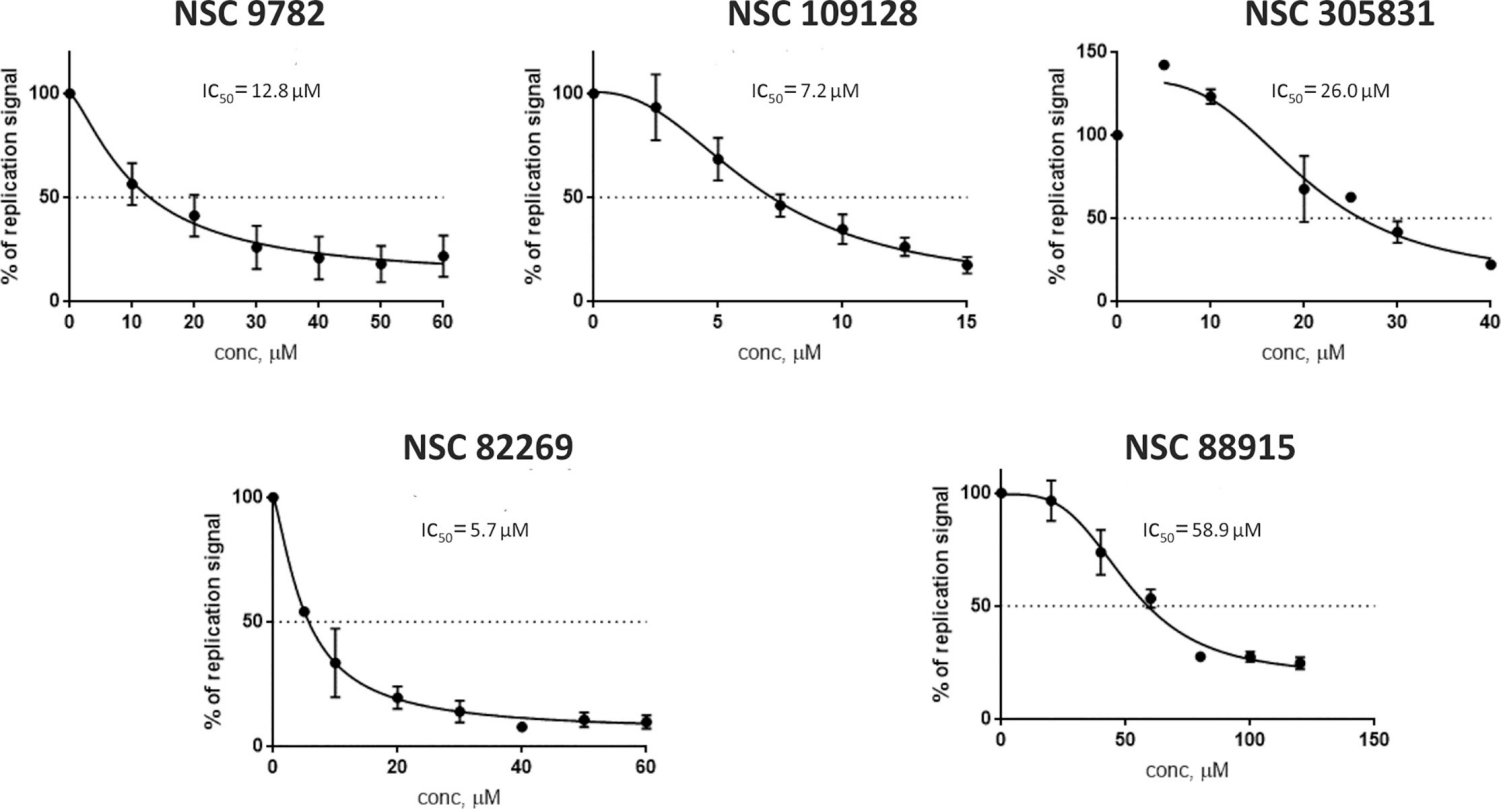
The inhibition of initial amplification of the MfPV8 genomes by the high-risk HPV-specific compounds. U2OS cells were transfected with 4 μg of the MfPV8 minicircle genomes and grown with the compounds for 5 days at the indicated concentrations. Genomic DNA was extracted, MfPV8 DNA was linearized and treated with DpnI, and analyzed by Southern blotting. The DNA replication signals of the MfPV8 were quantified (ImageQuant TL 8.1, GE Healthcare), expressed relative to the vehicle control (DMSO), and the inhibition curves and IC50 values for each compound were calculated by curve fitting ([inhibitor] vs. concentration, variable slope, 4 parameters). Error bars represent the standard deviation from 2–3 independent experiments.

MfPV8 is associated with CIN [23], and its gene expression pattern is characteristic of the high-risk mucosal HPV subtypes during transient replication (S2 Fig). Thus, we decided to analyze whether the previously described high-risk HPV-specific drug candidates can also inhibit MfPV8 replication. The five compounds used (NSC 9782, NSC 88915, NSC 82269, NSC 109128 and NSC 305831) were identified in high-throughput screening of NCI Diversity Set IV chemical library as specific inhibitors of high-risk mucosal HPV replication [32]. The MfPV8 minicircle genomes were transfected into U2OS cells, and genomic DNA was harvested after cultivating the cells in the presence of each compound at the concentrations for 5 days. The extracted DNA was treated with DpnI and the linearizing enzyme and subjected to Southern blot analysis. The DpnI-resistant replication signal strengths were quantified and expressed relative to the vehicle-only control (DMSO), and the inhibition curves and IC50 values for each compound are provided in Fig 4. The results indicate that all five compounds inhibited the replication intensity of the MfPV8 genomes in a concentration-dependent manner, with IC50 values ranging from 5.7 to 58.9 μM (Fig 4), which are very similar to what is seen for HPV18, for example [32]. Thus, MfPV genome replication in U2OS cells can potentially be used for screening or validation of anti-HPV drugs.

## Discussion

While cervical cancer is one of the leading causes of cancer mortality among women worldwide, no adequate animal models accurately mimicking PV infection in humans currently exist for testing the safety and efficacy of the compounds identified as HPV drug candidates. The genus *Alphapapillomavirus* is medically important, containing the oncogenic genital PV types being responsible for nearly all cases of human cervical cancer and a number of anal, penile, and head and neck carcinomas [8,9]. Alpha-PVs have also been identified in nonhuman primates, causing obvious clinical symptoms similar to PV-associated lesions observed in humans [1,21–25]. Different cynomolgus macaque PVs identified from the cervicovaginal area cluster within the alpha 12 species and show a close phylogenetic relationship with high-risk HPVs (e.g., HPV16) [1,23,25]. Additionally, a cutaneous type has been identified in severe papillomas on the hands and feet of a cynomolgus macaque, and it clustered with HPV5 into the genus β-PVs, to which all EV-type PVs belong [21]. Notably, similarities between specific MfPV and HPV types are detected at the sequence level and genome organization; however, all these findings have been reported based on sequence analyses [21,23].

Interestingly, we found that the human U2OS cell line is capable of supporting the replication of not only HPV [26–32,36,42] but also cynomolgus macaque PV genomes (Fig 1) and therefore provides an opportunity to characterize these viruses, which are closely related to HPVs, at the molecular level. Hence, we used the U2OS cell line-based assay system for experimental description of the high-risk alpha and beta MfPV transcriptomes. We demonstrated that both analyzed mucosal MfPV5 and 8 predominantly employed the early promoter region within the LCR, and the TSS mapped immediately upstream of the E6 gene (at nt 7982 and 7996, respectively) (S1 and S2 Figs), which is consistent with those described in several high-risk HPVs, such as P97 for HPV16 [43,44] and P102 for HPV18 [29,35,45–47]. Additional TSSs positioning in the E6, E7, E1 and E2 ORFs were detected (marked in S1 and S2 Figs), and similar promoter regions have also been described for HPV18 [29,45]. The beta-PV type MfPV1 showed more heterogeneity in TSS usage, and a strong promoter region with multiple TSSs in the E7 ORF was observed (S3 Fig). This type of TSS usage has also been observed for beta HPV type 5 by Sankovski and others [30]. In addition to selective promoter usage, viral transcripts can undergo extensive alternative splicing, which results in a very complex transcription map. The identified splicing donor and acceptor sites of the MfPV5 and 8 genomes (marked in S1 and S2 Figs) share clear sequence homology with the corresponding SD and SA sites of human high-risk types 16 and 18 [29,35,43,48]. The same observations can be noted between the SD and SA regions of MfPV1 (marked in S3 Fig) and closely related HPV5 [30]. For the most part, only oncogenic mucosal HPVs (e.g., HPV16, 18, 31, 45) have a splice donor and acceptor pair within the E6 ORF that enable the expression of a shorter internally spliced version of the E6 proteins (termed E6*), facilitating more effective translation of the E7 oncoprotein [49–51]. The sequence analysis of the MfPV5 genome revealed the presence of this canonical SD site within the E6 ORF [23], and our transcriptome mapping experiments confirmed those results (S1 Fig, RNAs B-D, G). Interestingly, regardless of the association of MfPV8 with CIN lesion [23], a mutation in the core motif of the SD site (AG^GT → AT^GT) within the E6 ORF is described (GenBank ID EF558842) [23]; indeed, we did not detect transcripts spliced inside of the E6 ORF (S2 Fig, RNAs A-D). Nevertheless, based on the sequence analysis, the MfPV8 oncoproteins seem to have characteristic features of high-risk HPVs. For instance, MfPV8 possesses a putative PDZ-binding motif in the carboxy terminus of the E6 protein [23] that is a unique characteristic of oncogenic HPVs (e.g., HPV16, HPV18) but absent in low-risk HPV E6s (e.g., HPV6, HPV11) [73]. It has been shown that E6 interacts with host proteins involved in cell signaling and adhesion through the C-terminal PDZ-binding motif and leads to their degradation (reviewed 70). The polyadenylation CSs of early mRNAs of all three MfPVs localized near the beginning of the L2 ORF (approximately nt 4310 for MfPV5, nt 4300 for MfPV8, and nt 4185 for MfPV1) (S1–S3 Figs, respectively) and exhibited sequence homology to closely related HPVs [29,30,35].

The transcriptome mapping experiments revealed several transcripts with the capability to encode viral replication proteins E1 and E2 (S1–S3 Figs). The implication of E1 and E2 proteins in the initiation of papillomavirus transient DNA replication is a thoroughly described mechanism [37,59–64]. For all three studied MfPV types, we demonstrated that the cotransfection of E1 and E2 mutant genomes, which are incapable of replication independently (Fig 2A–2C, lanes 4–9), resulted in restored replication competence of both genomes (Fig 2A–2C, lanes 10–12). This clearly indicates that two defective viral genomes complemented each other’s defects, suggesting that the initial amplification of the MfPV genomes is strictly dependent on the E1 and E2 proteins.

In addition to the viral E2 full-length protein, PV genomes also encode the truncated forms of E2 proteins that modulate viral gene expression. The E8^E2 protein has been shown to be the major negative regulator limiting PV transcription and inhibiting E1- and E2-dependent viral DNA replication [52–54]. This type of copy number regulation has been previously identified in several human and animal papillomaviruses [52–57]. This was also confirmed by our results that the E8 mutant genomes of MfPV1, 5, and 8 exhibited significantly higher replication levels compared to wild-type genomes (Fig 2A–2C, compare lanes 1–3 and 13–15). The effect was most striking for cutaneous MfPV1, i.e., wild-type genomes show rather modest replication signals that even decreased at later time points, while very efficient time-dependent viral DNA replication was notable for the E8 mutant genomes (Fig 2C, lanes 1–3 and 13–15). In fact, most human cell lines are not capable of supporting full-length HPV genome replication; nevertheless, the replication of papillomavirus origin-containing plasmids can be readily accomplished in many cell lines by expressing the viral replication proteins E1 and E2 from heterologous vectors [59,74]. Thus, it would be interesting to analyze whether E8^E2 protein-mediated replication suppression plays a role in restricting HPV genome replication in certain cell types. Although transcriptome analyses revealed two E8-ORF-containing transcripts (E8^E1 and E8^E2 mRNAs) (S1–S3 Figs, mRNAs J and K, G and H, D and E, respectively), this effect is most likely due to the loss of E8^E2 expression because the E8^E1 protein seems to have no significant role in affecting viral copy number and transcription from the URR region of several HPVs [29–31].

HPV tends to form genomic oligomers during the initial amplification phase, and these larger DNA molecules are formed through the homologous recombination of replicating molecules [27]. It has been shown for HPV18 that the prevalence of oligomeric genomes increases over time, and during the stable maintenance phase, only the higher-molecular-weight forms can be detected [26,27]. This suggests that genomic oligomers likely have a selective advantage over monomeric viral DNA forms during the stable maintenance replication phase. Additionally, multimeric papillomavirus genomes have also been detected in HPV-infected keratinocyte cell lines [27,70,71], BPV1-transformed mouse fibroblast cell line ID13 [72], and clinical samples obtained from HPV-associated cervical lesions [27,65–69]. Studying the molecular state of the MfPV genomes during the initial amplification phase in U2OS cells, we noticed effective initiation of viral DNA replication in the case of all three studied MfPVs, and in addition to the extrachromosomal circular monomeric genomes, dimeric and oligomeric forms also appeared in a time-dependent manner (Fig 3). This suggests that the multimerization of viral genomes is a common process during HPV infections in vivo, and our results clearly demonstrate that this is also a characteristic feature of MfPV genome replication.

Moreover, the fact that the replication of the MfPV8 genomes is sensitive to high-risk HPV-specific compounds (Fig 4) indicates similarities in the replication properties of the high-risk mucosal MfPV and HPV genomes. All five tested compounds were identified in high-throughput screening of the NCI Diversity Set IV chemical library [32], and two of them (NSC 88915 and NSC 305831) were previously described as tyrosyl-DNA-phosphodiesterase type 1 (Tdp1) inhibitors [75,76]. Tdp1 is a DNA damage response network protein involved in releasing entrapped topoisomerases from DNA lesions [77]. The tested compounds are considered specific high-risk mucosal HPV inhibitors since these are not effective against cutaneous beta (HPV5) and low-risk mucosal (HPV11) HPV DNA replication [32]. It is likely that there might be differences in pathways involved in mucosal low- and high-risk HPV DNA replication, indicating that a relevant in vivo model that adequately mimics the molecular mechanisms characteristic of specific HPVs is an important prerequisite for the preclinical testing of HPV drug candidates.

MfPVs are relatively common natural pathogens in cynomolgus macaques in which PV-induced cervical neoplasia occurs quite frequently. Even the experimental transmission of MfPV3 from a naturally infected female to naïve female macaques initiated CIN development [78]. The progression and morphology of these lesions in macaques share distinctive similarities with those observed in women [78,79]. Moreover, high-grade lesions have been frequently observed at the squamocolumnar junction where most HPV-induced cervical cancers arise [79]. Despite the fact that this cell culture-based research has limitations (e.g., lack of ability to study viral immortalization or transformation potential, focusing only on the viral initial amplification phase), our findings, together with above-mentioned observations, could justify more physiologically relevant studies in *Macaca fascicularis* to further develop a relevant in vivo model. Since such animal model provides an extremely important resource for therapeutic development, it merits further investigation.

Although PVs are considered strictly species-specific viruses, it is noteworthy that some macaque PVs exhibit cross-species infectivity identified in both rhesus and cynomolgus macaques [23,78,80]. Interestingly, a human skin wart has been successfully transmitted to the lower genital tract of a male chimpanzee (*Papio papio*). Nevertheless, there is possibility that transmitted lesions were not induced by viral infection but resulted from heterotransplantation of human papilloma cells; however, it is believed to be rarely successful among higher animals [81,82]. In vitro studies demonstrate that HPV16 successfully maintains an extrachromosomal episome in monkey kidney cells [83], and conversely, our findings indicate that the human U2OS cell line supports the DNA replication of the MfPV genomes. Thus, HPVs may potentially establish persistent infection in cynomolgus macaques. Alternatively, to viral infection, naked HPV genomic DNA transfer using chemical transfection or epidermal electroporation [84] could be used for delivery of the viral genomic DNA into the proliferating epidermal keratinocytes of cynomolgus macaques. This should allow establishing the presence and, expectantly, the replication of viral genomes in the target tissue.

## Supporting information

S1 Fig Summary of the MfPV5 transcripts that were mapped in transiently transfected U2OS cells. At the top, the schematic depiction of the linear early region of the MfPV5 genomes with the ORFs, LCR, the predominant and less frequently used TTSs (indicated with arrows), and polyadenylation CSs (indicated with arrowheads) are shown. The TSS data were collected from the sequences of a total of 57 clones of 5’ RACE products. The defined E1^E4 and E8^E2 mRNAs span over two exons are also indicated. All mRNA species experimentally identified here (marked with letters) are represented with exons (solid boxes), introns (lines), and mapped splicing donor and acceptor sites. The coding potential of each transcripts is displayed on the right. The RNA spices identified by RT-PCR and without exact TSS mapped are depicted with inferred 5′ ends (RNAs J and K). The numbers indicate nucleotide position within the viral genome.
(TIF)

S2 Fig Summary of the MfPV8 transcripts that were mapped in transiently transfected U2OS cells. At the top, the schematic depiction of the linear early region of the MfPV8 genomes with the ORFs, LCR, the predominant and less frequently used TTSs (indicated with arrows), and polyadenylation CSs (indicated with arrowheads) are shown. The TSS data were collected from the sequences of a total of 38 clones of 5’ RACE products. The defined E1^E4 and E8^E2 mRNAs span over two exons are also indicated. All mRNA species experimentally identified here (marked with letters) are represented with exons (solid boxes), introns (lines), and mapped splicing donor and acceptor sites. The coding potential of each transcripts is displayed on the right. The RNA spices identified by RT-PCR and without exact TSS mapped are depicted with inferred 5′ ends (RNAs G and H). The numbers indicate nucleotide position within the viral genome.
(TIF)

S3 Fig Summary of the MfPV1 transcripts that were mapped in transiently transfected U2OS cells. At the top, the schematic depiction of the linear early region of the MfPV1 genomes with the ORFs, LCR, the predominant and less frequently used TTSs (indicated with arrows), and polyadenylation CSs (indicated with arrowheads) are shown. The TSS data were collected from the sequences of a total of 37 clones of 5’ RACE products. The defined E1^E4 and E8^E2 mRNAs span over two exons are also indicated. All mRNA species experimentally identified here (marked with letters) are represented with exons (solid boxes), introns (lines), and mapped splicing donor and acceptor sites. The coding potential of each transcripts is displayed on the right. The RNA spices identified by RT-PCR and without exact TSS mapped are depicted with inferred 5′ ends (RNAs D and E). The numbers indicate nucleotide position within the viral genome.
(TIF)

S1 Table The transcripts identified by 5’ RACE or RT-PCR analyses. (XLSX)

S2 Table The transcripts identified by 3’ RACE analysis. (XLSX)
