# Supplementary figures and images for: The molecular biology and HPV drug responsiveness of cynomolgus macaque papillomaviruses support their use in the development of a relevant in vivo model for antiviral drug testing
Source: PLoS One. 2019 Jan 25;14(1):e0211235. doi: 10.1371/journal.pone.0211235 (PMC6347367; doi:10.1371/journal.pone.0211235)

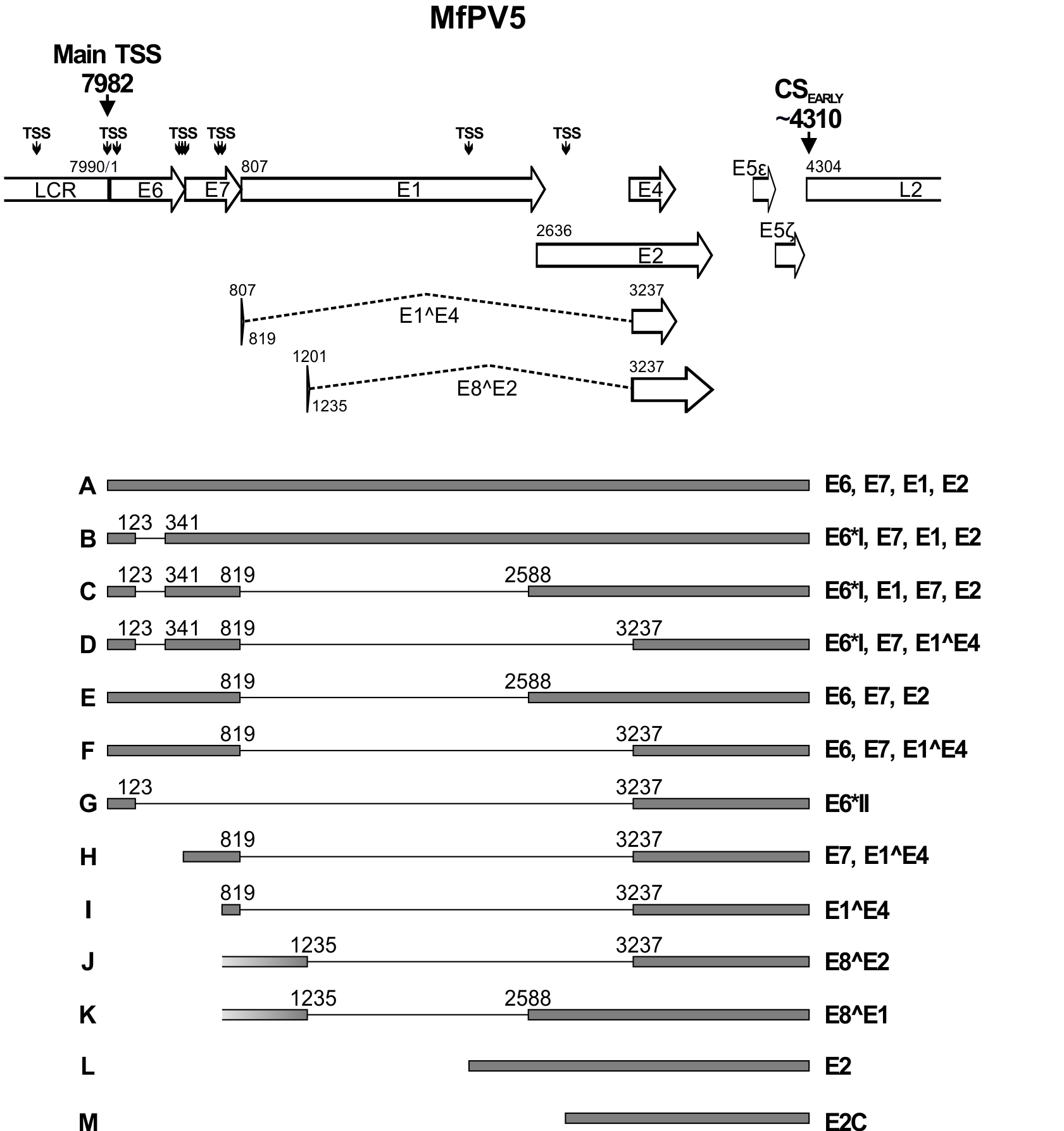

Supplement: S1 Fig — At the top, the schematic depiction of the linear early region of the MfPV5 genomes with the ORFs, LCR, the predominant and less frequently used TTSs (indicated with arrows), and polyadenylation CSs (indicated with arrowheads) are shown. The TSS data were collected from the sequences of a total of 57 clones of 5’ RACE products. The defined E1^E4 and E8^E2 mRNAs span over two exons are also indicated. All mRNA species experimentally identified here (marked with letters) are represented with exons (solid boxes), introns (lines), and mapped splicing donor and acceptor sites. The coding potential of each transcripts is displayed on the right. The RNA spices identified by RT-PCR and without exact TSS mapped are depicted with inferred 5′ ends (RNAs J and K). The numbers indicate nucleotide position within the viral genome. (TIF) [file pone.0211235.s001.tif]

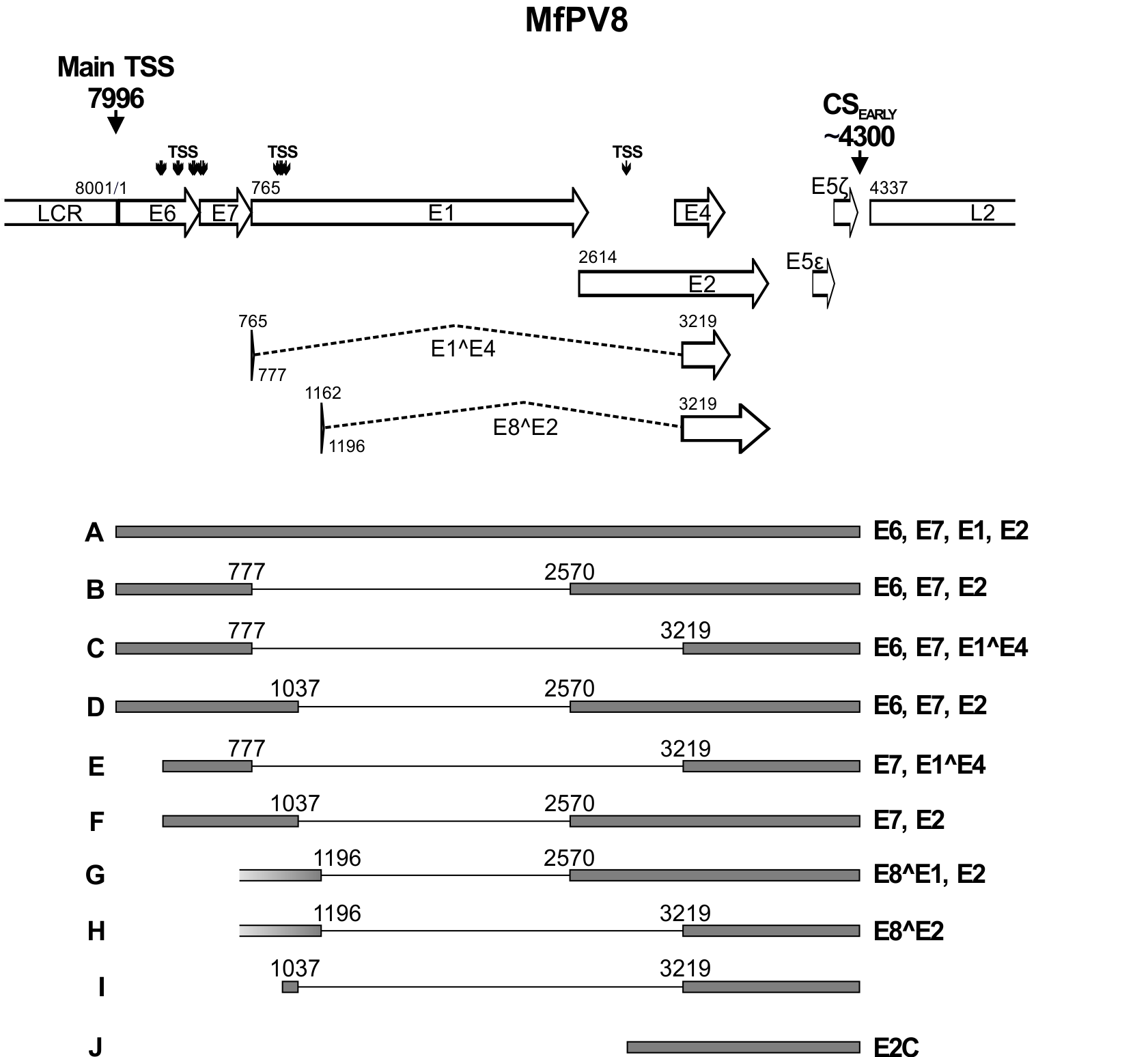

Supplement: S2 Fig — At the top, the schematic depiction of the linear early region of the MfPV8 genomes with the ORFs, LCR, the predominant and less frequently used TTSs (indicated with arrows), and polyadenylation CSs (indicated with arrowheads) are shown. The TSS data were collected from the sequences of a total of 38 clones of 5’ RACE products. The defined E1^E4 and E8^E2 mRNAs span over two exons are also indicated. All mRNA species experimentally identified here (marked with letters) are represented with exons (solid boxes), introns (lines), and mapped splicing donor and acceptor sites. The coding potential of each transcripts is displayed on the right. The RNA spices identified by RT-PCR and without exact TSS mapped are depicted with inferred 5′ ends (RNAs G and H). The numbers indicate nucleotide position within the viral genome. (TIF) [file pone.0211235.s002.tif]

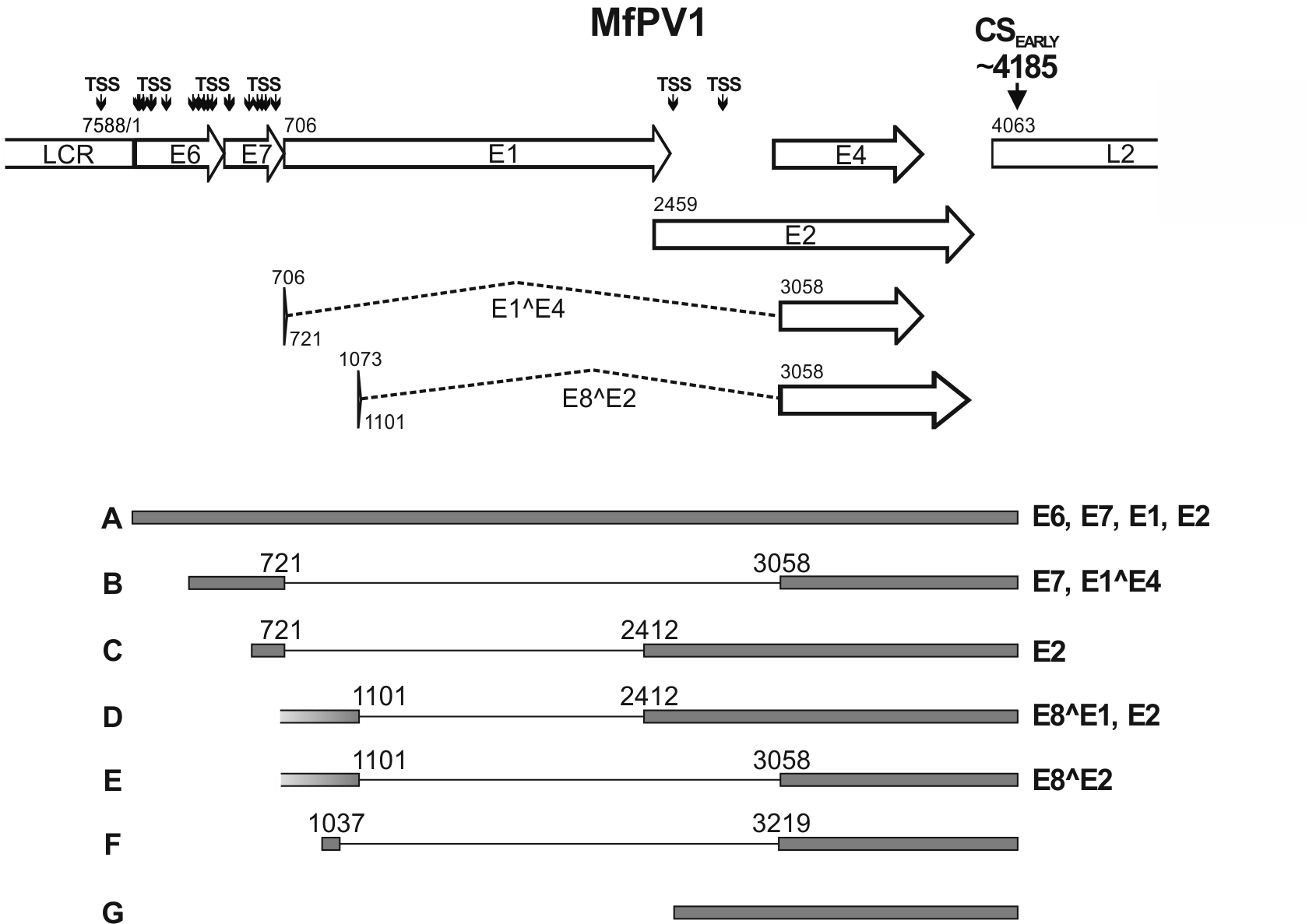

Supplement: S3 Fig — At the top, the schematic depiction of the linear early region of the MfPV1 genomes with the ORFs, LCR, the predominant and less frequently used TTSs (indicated with arrows), and polyadenylation CSs (indicated with arrowheads) are shown. The TSS data were collected from the sequences of a total of 37 clones of 5’ RACE products. The defined E1^E4 and E8^E2 mRNAs span over two exons are also indicated. All mRNA species experimentally identified here (marked with letters) are represented with exons (solid boxes), introns (lines), and mapped splicing donor and acceptor sites. The coding potential of each transcripts is displayed on the right. The RNA spices identified by RT-PCR and without exact TSS mapped are depicted with inferred 5′ ends (RNAs D and E). The numbers indicate nucleotide position within the viral genome. (TIF) [file pone.0211235.s003.tif]
